# Polydopamine-infused toothpaste: An in vitro assessment of cytotoxicity and embryonic toxicology

**DOI:** 10.1016/j.jobcr.2025.09.005

**Published:** 2025-09-08

**Authors:** Ayesh Das, Jayashri Prabakar, I. Meignana Arumugham, S. Rajeshkumar, Jayasree Anandan, Amal Siby

**Affiliations:** a Department of Public Health Dentistry, Saveetha Dental College, Saveetha Institute of Medical and Technical Sciences [SIMATS], Saveetha University, Chennai, 600077, Tamil Nadu, India; b Nanobiomedicine Lab, Centre for Global Health Research, Saveetha Medical College and Hospitals, Saveetha Institute of Medical and Technical Sciences, Saveetha University, Chennai, 602105, Tamil Nadu, India; c Healthcare Management, York St John University, London, United Kingdom

**Keywords:** Polydopamine, Therapeutic toothpaste, Oral care, Cytotoxicity, Antibacterial activity, Biocompatibility

## Abstract

**Background:**

Oral health issues affect approximately 3.5 billion individuals globally, with untreated dental caries being the most prevalent condition, as reported by the WHO. While mechanical plaque removal has shown effectiveness in preventing oral diseases, the need for more advanced and bioactive toothpaste formulations persists. Among emerging ingredients, polydopamine (PDA) has shown promising antibacterial and biocompatible properties, making it a potential additive for therapeutic oral care products.

**Aim:**

This study aimed to formulate a polydopamine-infused therapeutic toothpaste and evaluate its in vitro cytotoxicity and embryonic toxicity.

**Methods:**

The toothpaste was developed using calcium carbonate as an abrasive, glycerin for moisture retention, and carboxymethyl cellulose as a thickening agent. Sodium lauryl sulfate functioned as a foaming agent, and sodium fluoride was included for enamel protection. Xylitol and peppermint oil were added to enhance taste and stability. Polydopamine was synthesized by polymerizing dopamine in an ethanol-water-ammonium hydroxide mixture and incorporated for antimicrobial enhancement. Cytotoxicity was assessed using the Brine Shrimp Lethality Bioassay, exposing *Artemia salina* nauplii to PDA-formulated toothpaste at concentrations ranging from 10 to 50 μg/mL. Embryonic toxicity was evaluated using the Zebrafish Embryo Toxicity Test over a 78- hour period. Statistical analysis involved Mann-Whitney and Friedman tests.

**Results:**

PDA-formulated toothpaste showed significantly lower cytotoxicity than commercial alternatives at all tested concentrations (p < 0.05), with no significant differences in embryonic toxicity. Both formulations exhibited dose-dependent responses (p = 0.001).

**Conclusion:**

Polydopamine-infused toothpaste showed reduced cytotoxicity and no embryonic toxicity, indicating its potential as a safe, effective therapeutic oral care additive.

## 1 Introduction

Tooth decay and periodontal diseases are among the most prevalent chronic conditions globally, with untreated dental caries affecting nearly 2 billion people.^1^ Fluoride-containing toothpaste remains the mainstay for dental caries prevention, offering substantial protection by enhancing enamel resistance and inhibiting demineralization.^2^ Yet, ongoing challenges in formulating toothpastes with enhanced antimicrobial and regenerative properties have driven research toward novel bioactive agents.^3^ Polydopamine (PDA), a biomimetic polymer inspired by mussel adhesion, has gained increasing interest in biomedical and dental fields due to its versatile bio functional properties, including strong adhesion, biocompatibility, and antimicrobial activity.^4^ Unlike traditional ingredients like triclosan or chlorhexidine, PDA provides multiple biological benefits without reported systemic toxicity.^5^

PDA has shown potential in promoting dentin remineralization by forming hydroxyapatite-like layers, which seal dentinal tubules and may reduce hypersensitivity.^6^ In vitro studies have confirmed its inhibitory action against *Streptococcus mutans*, the primary pathogen associated with dental caries.^7^ Additionally, PDA-modified hydrogels have demonstrated efficacy in soft and hard tissue regeneration in dental applications, owing to their enhanced adhesive and osteoconductive properties^8^

The rationale for incorporating polydopamine into toothpaste lies in its unique combination of antimicrobial, demineralizing, and tissue-compatible characteristics, which may synergistically improve oral health outcomes. While the use of PDA in dental composites, membranes, and coatings has been previously reported,^9^ limited data are available regarding its direct application in dentifrice formulations. Moreover, safety evaluation, particularly related to cytotoxicity and embryonic toxicity, remains an essential step before clinical translation.^10^ Recent studies have also shown that PDA-coated polyhydroxyalkanoate (PHA) films improve tissue integration, enhance fibroblast attachment and proliferation, and exhibit better *in vivo* biocompatibility compared to uncoated materials.^11^^,^^12^

Given these promising properties, the current study aims to formulate a toothpaste incorporating polydopamine as a core active ingredient. The objective is to evaluate this novel formulation for its biological safety and effectiveness by conducting in vitro assessments of its cytotoxic potential and embryonic toxicity. This study represents a step toward expanding the scope of therapeutic agents in oral care products to develop multifunctional, bioactive toothpaste formulations that offer enhanced protection against oral diseases.

## 2 Materials and methodology

### 2.1 Materials

All chemicals for preparing the base of the toothpaste were bought from SRL CHEMICALS. For preparing polydopamine, Dopamine was brought from Chemical otto.

### 2.2 Study setting

The research laboratory of Saveetha Institute of Medical and Technical Science formulated a novel toothpaste, a novel polydopamine-infused toothpaste and studied it in vitro for cytotoxicity assessment. The study was conducted in January 2024.

### 2.3 Preparation of toothpaste

The formulation of the toothpaste involved combining essential ingredients commonly used in standard toothpaste compositions. Abrasives such as calcium carbonate were utilized to aid in the mechanical removal of plaque and surface stains. Humectants like glycerin were incorporated to retain moisture and prevent the paste from drying. Carboxymethyl cellulose served as a binder to maintain consistency, while sodium lauryl sulfate acted as a foaming agent to facilitate the dispersion of the paste during brushing. Fluoride compounds, specifically sodium fluoride, were included to enhance enamel strength and provide anticaries benefits. Additionally, flavoring agents such as peppermint oil, sweeteners like xylitol, preservatives, and coloring agents were carefully added to enhance the overall acceptability and stability of the product.^13^

**Fig. 1:**
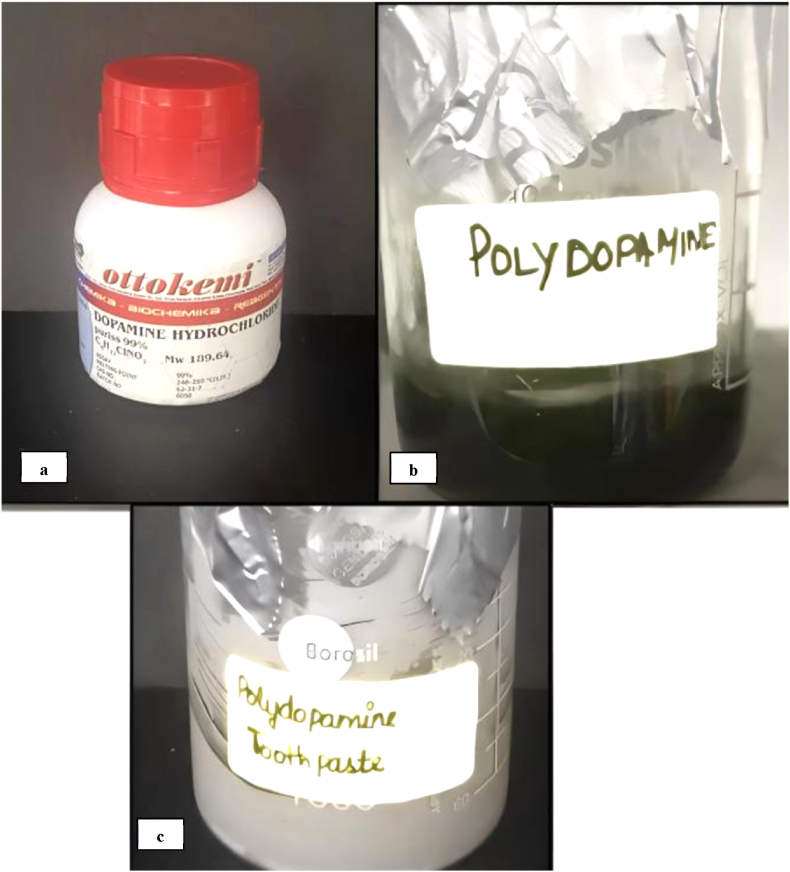
Showing: a. Dopamine Hydrochloride, b. Polydopamine and c. Polydopamine toothpaste.

To enhance the functional properties of the formulation, polydopamine was synthesized separately. At 25 °C, 50 mg of dopamine (CHEMICAL OTTO) was dissolved in 1 ml of deionized water. This solution was then added to a pre-stirred mixture (MAGNETIC STIRRED, REMI) containing 5 ml of ethanol, 9 ml of water, and 0.15 ml of ammonium hydroxide (AS). The initially clear solution rapidly turned pale brown and progressively darkened to a deep brown, indicating the formation of polydopamine nanoparticles (Fig. 1). The reaction mixture was stirred continuously and allowed to proceed for 24 h to ensure complete polymerization. The prepared polydopamine was later incorporated into the toothpaste formulation to confer additional antimicrobial and surface-adhering properties**.** The procedure was carried out with slight modifications, outlined in the previous study conducted by Rahila et al.^14^**.**

### 2.4 Cytotoxic assessment

#### 2.4.1 Brine Shrimp Lethality Bioassay^15^

**Fig. 2:**
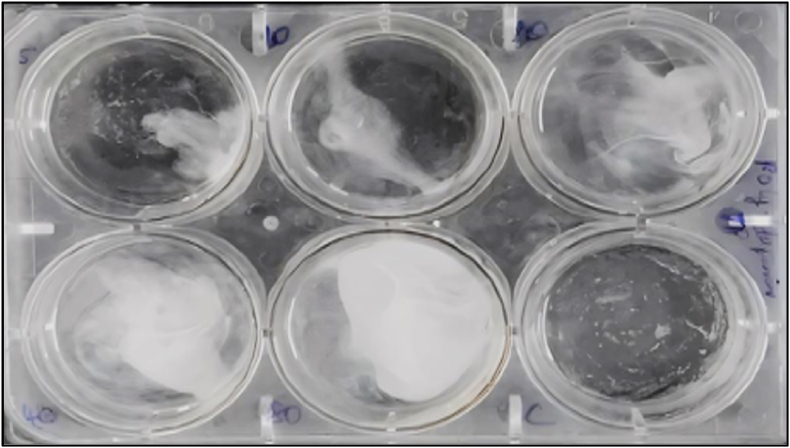
Brine Shrimp Lethality Assay for testing cytotoxic assessment.

To perform the assay, 2 g of iodine-free salt were dissolved in 200 mL of distilled water to create a saline solution. Using six-well ELISA plates, approximately 10–12 mL of the saline was added to each well. Ten brine shrimp larvae (nauplii) were carefully introduced into each well (Fig. 2). The test groups received toothpaste formulations containing polydopamine at concentrations of 10, 20, 30, 40, and 50 μg/ml. The setup was incubated for 24 h under controlled conditions. After the incubation period, the number of surviving nauplii in each well was counted. The mortality rate was determined using the following formula:

Mortality(%)=(Numberofdeadnauplii/Totalnumberofnauplii)×100

The results were documented accordingly, and visual data were compiled.

### 2.5 Embryotoxicity Assessment^16^

#### 2.5.1 Zebrafish Embryo Toxicity Test

**Fig. 3:**
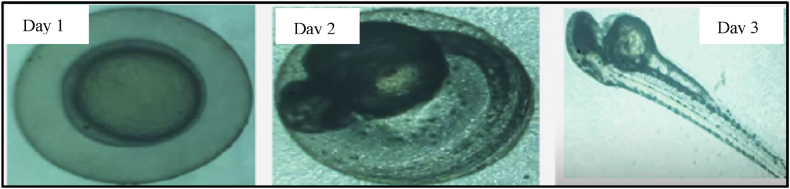
Zebrafish embryo evaluation for testing embryonic toxicology.

Zebrafish embryos were observed under a stereo microscope from fertilization to assess developmental progress. They were exposed to polydopamine at concentrations of 5, 10, 20, 40, and 80 μg/mL for 24–78 h post-fertilization. After this exposure, polydopamine was introduced at concentrations of 10, 20, 30, 40, and 50 μg/mL to evaluate its potential embryotoxic effects. (Refer Fig. 3).

Embryos were observed at 24-h intervals to monitor key developmental milestones, including survival rates, hatching success, and the presence of any physical abnormalities. Both control and experimental groups were compared. Any deformities in embryos and larvae were documented, and representative images were captured using a COSLAB HL-10A microscope. The percentage of malformations was calculated at each 24-h checkpoint throughout the exposure period.

### 2.6 Statistical analysis

All experiments were conducted in triplicate. The Mann–Whitney *U* test was used to compare cytotoxicity and embryonic toxicology between the PDA toothpaste and a commercially available fluoride toothpaste. The Friedman test, a non-parametric repeated measures test, was used to analyze concentration-dependent effects within each treatment group. A p-value <0.05 was considered statistically significant. Data were analyzed using SPSS version 26.0 (IBM Corp., Armonk, NY, USA).

## 3 Results

### 3.1 Cytotoxic assessment

The comparative cytotoxic assessment between polydopamine and commercial toothpaste (Table 1) demonstrated statistically significant differences at all tested concentrations, as determined by the Mann-Whitney test (P < 0.05 for all comparisons). At each concentration (10–50 μg/ml), commercial toothpaste exhibited consistently higher cytotoxicity values than polydopamine, with mean differences ranging from approximately 5 μg/ml. The lower cytotoxicity associated with polydopamine suggests a comparatively better biocompatibility profile than commercial toothpaste. These findings highlight that, while both formulations exhibit a concentration-dependent increase in cytotoxicity, polydopamine presents as a potentially safer alternative for incorporation into oral care products, warranting further investigation and development.

**Table 1:** Comparison of the mean cytotoxicity of polydopamine and commercial toothpaste across and within various concentrations.

Concentration (μg/ml) (mean ± SD)	Polydopamine (μg/ml) (mean ± SD)	Commercial Toothpaste (μg/ml) (mean ± SD)	Mann-Whitney U test- P value
**10**	50.8 ± 0.83	55.6 ± 1.51	0.008∗
**20**	61.8 ± 1.48	67.8 ± 1.48	0.001∗
**30**	67.2 ± 1.30	74.1 ± 1.64	0.008∗
**40**	73.6 ± 1.14	81.2 ± 0.83	0.008∗
**50**	78.8 ± 1.48	86.8 ± 1.48	0.009∗
**Friedmann test P value**	0.001∗		

The cytotoxic effects of Polydopamine and Commercial Toothpaste across concentrations ranging from 10 to 50 μg/ml. Both materials demonstrated a concentration-dependent increase in cytotoxicity. For Polydopamine, the mean cytotoxicity values increased from 50.8 ± 0.83 at 10 μg/ml to 78.8 ± 1.48 at 50 μg/ml. In contrast, Commercial Toothpaste exhibited higher mean cytotoxicity values, ranging from 55.6 ± 1.51 at 10 μg/ml to 86.8 ± 1.48 at 50 μg/ml. Notably, at every tested concentration, Commercial Toothpaste showed greater cytotoxicity compared to Polydopamine. Furthermore, the standard deviations were slightly higher for Commercial Toothpaste, suggesting marginally greater variability in response. Both groups showed statistically significant differences across concentrations, confirmed by Friedman test results with P values of 0.001. Overall, these results indicate that while both materials display a significant and concentration-dependent cytotoxic response, Commercial Toothpaste exhibits a consistently higher level of cytotoxicity than Polydopamine across all tested concentrations.

### 3.2 Embryonic toxicology

The embryonic toxicology comparison between polydopamine and commercial toothpaste (Table 2) showed no statistically significant differences across all tested concentrations (10–50 μg/ml), as indicated by the Mann-Whitney test (P > 0.05 for all comparisons). Although the commercial toothpaste consistently exhibited slightly higher mean values compared to polydopamine, the differences were minimal and statistically nonsignificant. This suggests that both polydopamine and commercial toothpaste exhibit a comparable level of embryonic toxicity within the tested concentration range. The findings support the embryonic biocompatibility of polydopamine, reinforcing its potential suitability for incorporation into oral health formulations without posing additional embryotoxic risks compared to conventional commercial products.

**Table 2:** Comparison of the mean Embryonic Toxicology of polydopamine and commercial toothpaste.

Concentration (μg/ml) (mean ± SD)	Polydopamine (μg/ml) (mean ± SD)	Commercial Toothpaste (μg/ml) (mean ± SD)	Mann-Whitney U test- P value
10	36.2 ± 1.30	38.6 ± 16.02	0.115
20	47.8 ± 1.92	51.0 ± 17.34	0.115
30	620.8 ± 0.8	65.8 ± 18.34	0.114
40	69.40 ± 0.89	71.60 ± 16.45	0.112
50	75.60 ± 1.14	76.2 ± 14.66	0.115
**Friedmann test P value**	0.001∗		

A comparative analysis reveals the embryonic toxicological profiles of Polydopamine and Commercial Toothpaste at concentrations ranging from 10 to 50 μg/ml. Both substances exhibited a concentration-dependent increase in embryonic toxicity. For Polydopamine, the mean toxicity values rose from 36.2 ± 1.30 at 10 μg/ml to 75.6 ± 1.14 at 50 μg/ml, whereas for Commercial Toothpaste, values increased from 38.6 ± 16.02 to 76.2 ± 14.66 across the same concentration range. Although the final toxicity levels at 50 μg/ml were comparable between the two materials, the standard deviations for Commercial Toothpaste were notably higher across all concentrations, indicating greater variability in its toxicological response compared to Polydopamine. Statistical analysis using the Friedman test demonstrated a highly significant difference across concentrations for both materials, with P values of 0.001. These findings suggest that while both Polydopamine and Commercial Toothpaste exhibit significant embryonic toxicity at higher concentrations, Polydopamine shows a more consistent and predictable toxicological profile, whereas Commercial Toothpaste demonstrates greater variability, potentially reflecting a more heterogeneous formulation.

## 4 Discussion

The findings from our study align with previous research demonstrating the crucial role of oral health in the prevention of systemic diseases. In particular, our investigation into the cytotoxicity of polydopamine (PDA)-based toothpaste in comparison to commercial toothpaste shows promising results for polydopamine as a safer alternative for oral care products. Both materials exhibited a concentration-dependent increase in cytotoxicity, yet commercial toothpaste consistently showed higher cytotoxicity values across all tested concentrations, indicating a more harmful profile. These results are consistent with earlier studies that highlight the biocompatibility of polydopamine,^17^ suggesting its potential for safer oral health applications.

The reduced cytotoxicity in the polydopamine-based toothpaste is likely attributed to the intrinsic properties of polydopamine rather than the exclusion of fluoride or other common ingredients, as both formulations contained similar base components. PDA is known for its free radical scavenging capacity, surface passivation, and biocompatibility, all of which contribute to mitigating cellular damage and improving formulation stability.^18^^,^^19^ It may also stabilise reactive compounds within the formulation, reducing localized toxicity caused by surfactants or preservatives.

Polydopamine's relatively lower cytotoxicity is consistent with its use in various biocompatible applications, including drug delivery systems and tissue engineering^20^ Our study's cytotoxicity results support the growing body of evidence advocating for the incorporation of PDA-based materials into oral health products, given their favourable biocompatibility and minimal toxic effects when compared to conventional materials. This aligns with the work of other researchers who have investigated the advantages of polydopamine coatings in promoting cellular adhesion and proliferation while maintaining safety^21^

In contrast, while both polydopamine and commercial toothpaste exhibited minimal differences in embryonic toxicity, the higher variability observed with commercial toothpaste may reflect a more heterogeneous formulation, as suggested by previous reports on the unpredictable toxicological profiles of commercial dental products.^22^ Our study's embryonic toxicology findings suggest that polydopamine may be a safer alternative with more consistent and predictable effects on embryonic development.

The growing interest in polydopamine-based materials for oral health is supported by studies demonstrating their inherent antibacterial properties, which are critical in preventing the colonization of oral pathogens such as *Streptococcus mutans* and *Porphyromonas gingivalis*.^23^ PDA exhibits antibacterial activity through mechanisms such as disruption of bacterial adhesion and induction of oxidative stress in microbial cells.^24^ This antimicrobial activity, when incorporated into toothpaste formulations, provides dual functionality: mechanical cleansing combined with microbial protection, potentially enhancing oral health outcomes.

Another key aspect of oral health is remineralization, which helps preserve the integrity of tooth enamel. The ability of polydopamine to promote remineralization, demonstrated by its capacity to chelate calcium ions and facilitate hydroxyapatite formation, complements the role of traditional fluoride-based toothpastes in enamel protection. However, concerns over fluoride toxicity have led to the exploration of alternatives, and our findings suggest that PDA could play an essential role in demineralizing tooth enamel without the risks associated with excessive fluoride exposure^25^

Over the years, many anti-caries products^26^, ^27^, ^28^ have been developed to overcome the drawbacks of traditional fluoride,^29^ such as the risk of fluorosis and its limited effectiveness in people with high caries activity. Researchers are now turning to newer, more natural solutions, and one promising option is polydopamine-infused oral care products. Due to its excellent adhesive nature, safety, and ability to support natural enamel repair while reducing bacterial growth, polydopamine could strengthen teeth and offer longer-lasting protection. This makes it an exciting new tool for preventing cavities and improving overall oral health.

While the promising results from our study suggest that polydopamine-based materials may hold great promise for oral health applications, it is essential to emphasize that clinical translation requires thorough safety evaluations and large-scale clinical trials. The long-term efficacy and cost-effectiveness of PDA-based products must be carefully assessed to ensure their suitability for widespread use. Regulatory and manufacturing considerations will also play a significant role in the commercial success of these innovative oral care products.

Strengths of the study include the comparative assessment of two toothpastes with equivalent base compositions, allowing for a focused evaluation of the effect of polydopamine alone. The dual toxicity model—brine shrimp and zebrafish embryo bioassays—adds robustness by capturing both cytotoxic and developmental endpoints. The use of graded concentrations provides clarity on dose-dependent effects, which is vital for future formulation adjustments and risk assessment.

This study was limited to in vitro cytotoxicity assays and short-term embryonic toxicity evaluation using zebrafish models, which may not fully replicate the complex biological environment of the human oral cavity. The Brine Shrimp Lethality Bioassay and Zebrafish Embryo Toxicity Test, while established screening tools, do not provide information on chronic exposure effects, long-term biocompatibility, or potential systemic toxicity. Furthermore, the antimicrobial activity of the polydopamine-infused toothpaste was not correlated with biofilm formation or persistence in *in vivo* conditions. The study also did not assess clinical parameters such as plaque index, gingival health, or enamel remineralization potential. The sample size for biological assays was relatively small, and environmental variables (e.g., pH, saliva composition) present in the human mouth were not simulated. Future studies should include *in vivo* animal models, larger sample sizes, extended exposure periods, and randomized controlled clinical trials to validate safety, efficacy, and long-term stability under realistic oral conditions.

## 5 Conclusion

Polydopamine-infused toothpaste, synthesized through an eco-friendly method, showed excellent biocompatibility with oral cell lines, reduced cytotoxicity, and no embryonic toxicity, highlighting its potential as a safe and effective therapeutic oral care product.

## Patient's/guardian's consent

The Patient's/Guardian's Consent is not applicable for this study.

## Ethical approval

Not required.

## Sources of Funding

Sources of Funding: This research did not receive any specific grant from funding agencies in the public, commercial, or not-for-profit sectors.

## Declaration of competing interest

The authors declare that they have no known competing financial interests or personal relationships that could have appeared to influence the work reported in this paper.
